# Diagnosis—Its Importance

**Published:** 1886-05

**Authors:** 


ARTICLE III.

### DIAGNOSIS—ITS IMPORTANCE.

Early in our professional career we often met in council an aged physician who was very highly educated, and whose counsel, as to the treatment of disease, was highly prized, yet it is probable that, in his own practice, he often, if not generally, failed to know what ailed his patients. Perhaps his powers of observation were naturally defective, but let this be as it was, when the pathological condition of your patient was pointed out to him he would give to you good, sensible advice as to treatment. Noticing this physician's misfortune, awakened us to the great importance of correct diagnosis.

Some physicians, or so-called ones, however, succeed in gaining the confidence of communities, and in amassing wealth, without much information in diagnosis or in anything else. One that we knew made reputation by calling every little execrescence a cancer, and burning it out with crude potash prepared by himself, accompanying the processs of preparation with some mystic flourishes, such as breathing on the material nine times while his eyes were closed. It was claimed in his behalf that this enabled him to discriminate so as to avoid sound underlving tissue, and thus "eat" only the cancer, A wart, a wen, a whitlow, or whatnot, was a cancer in his vocabulary, and, that his medicine would eat it was holy-writ proof that his diagnosis was correct.

In like manner, we see, almost daily, a quack who calls any cough consumption, increased action of the kidneys, due to cooler weather, is Bright's disease, a common itch is scrofula, and eczema is leprosy; and, as the fool-killer has long been derelict in duty. he prospers financially.

By mistaking or mis-stating the diagnosis, a man often gets credit for marvelous, and almost miraculous cures.

Dentists don't treat such great varieties in disease as do physicians; but they too have their difficulties and make mistakes. The disease known as *pyorrhœa alveolaris* illustrates this truth. A patient's mouth may be very offensive, his gums may be red, spongy and swollen, crusts of salivary calculus may abound, and the dentist may call the trouble by the long latin name above, scrape off the tartar, apply a lotion, suggest a tooth-brush, and report the case cured by a single application. Are not such cases common? Does the description refer to a familiar or to an unfamiliar scene? But all named in the case above may be present, and all claimed may occur, and yet no *pyorrhœa alveolaris* has been cured, nor has it been even treated.

A genuine case of the virulent disease may present milder symptoms than are described above; yet the man who professes to cure genuine *pyorrhœa alveolaris* in a day, and by a single application, may be a good manipulative dentist, and an honest, sincere man, but we always take for granted that he has made a mistake in diagnosis. The man who really understands the pathology of this disease will not regard it as purely local, and he will not rely wholly on scraping, or other local measure.

In treating pyorrhœa it is quite important to remove all deleterious and unpleasant accumulations from the teeth, and even from the entire mouth. Cleanliness is akin to godliness; and it is as important here as in the treatment of typhoid fever. No sane physician would allow excrementitious matter to remain in the bedroom of the invalid in such case, but he would not claim to have cured the patient by its removal. And were the typhoid patient to regain his health within a day after a single removal, we would all know the physician had erred in diagnosis when he called it typhoid fever.

An eccentric physician claimed that he was making a good average in his practice, as he cured a goodly number who were getting well anyway, and killed some who would have died even without treatment. It may be that some of our specialty have had similar experience, even when endeavoring to do the best for his patients.

But if in writing this we succeed in calling the attention of our readers to the importance of correct diagnosis, thereby inducing some to greater carefulness and closer study, we shall be well repaid for the brief effort. In referring to the cases of extreme disregard in this direction it is not our purpose to intimate that we know of dentists deserving to be classed with such; but in setting forth extremes, we can better show the exceeding blackness that is possible when developed by the united forces of ignorance and dishonesty. —*Ohio State Journal of Dental Science.*

